# Consensus statements on PSMA PET/CT response assessment criteria in prostate cancer

**DOI:** 10.1007/s00259-020-04934-4

**Published:** 2020-07-02

**Authors:** Stefano Fanti, Karolien Goffin, Boris A Hadaschik, Ken Herrmann, Tobias Maurer, Steven MacLennan, Daniela E. Oprea-Lager, Wim JG Oyen, Olivier Rouvière, Nicolas Mottet, Anders Bjartell

**Affiliations:** 1 Nuclear Medicine Division, Policlinico S Orsola, University of Bologna, Bologna, Italy; 2 Department of Nuclear Medicine and Molecular Imaging, University Hospital Leuven and KU Leuven, Leuven, Belgium; 3 Department of Urology, University of Duisburg-Essen and German Cancer Consortium (DKTK)-University Hospital Essen, Essen, Germany; 4 Department of Nuclear Medicine, University of Duisburg-Essen and German Cancer Consortium (DKTK)-University Hospital Essen, Essen, Germany; 5 Department of Urology and Martini-Klinik Prostate Cancer Center, Universitätsklinikum, Hamburg-Eppendorf, Hamburg, Germany; 6 Academic Urology Unit, University of Aberdeen, Foresterhill, Aberdeen, UK; 7 Department of Radiology and Nuclear Medicine, Amsterdam University Medical Centers, VU University, Amsterdam, The Netherlands; 8 Humanitas University and Humanitas Clinical and Research Center, Milan, Italy; 9 Department of Radiology and Nuclear Medicine, Radboud University Medical Centre, Nijmegen, The Netherlands; 10 Department of Radiology and Nuclear Medicine, Rijnstate Hospital Arnhem, Arnhem, The Netherlands; 11 Department of Imaging, Hôpital Edouard Herriot, Hospices Civils de Lyon, Lyon, France; 12 Faculté de Médecine Lyon Est, Université de Lyon, Université Lyon 1, 69003 Lyon, France; 13 University Hôpital Nord, Saint Etienne, France; 14 Department of Urology, Skåne University Hospital, Malmö, Sweden

**Keywords:** Monitoring, Oligometastatic prostate cancer, PERCIST, PET/CT, Polymetastatic prostate cancer, Prostate cancer, Prostate-specific membrane antigen (PSMA), PSMA ligand, RECIST

## Abstract

**Purpose:**

Prostate-specific membrane antigen (PSMA) positron emission tomography (PET)/computed tomography (CT) is used for (re)staging prostate cancer (PCa) and as a biomarker for evaluating response to therapy, but lacks established response criteria. A panel of PCa experts in nuclear medicine, radiology, and/or urology met on February 21, 2020, in Amsterdam, The Netherlands, to formulate criteria for PSMA PET/CT-based response in patients treated for metastatic PCa and optimal timing to use it.

**Methods:**

Panelists received thematic topics and relevant literature prior to the meeting. Statements on how to interpret response and progression on therapy in PCa with PSMA PET/CT and when to use it were developed. Panelists voted anonymously on a nine-point scale, ranging from strongly disagree (1) to strongly agree (9). Median scores described agreement and consensus.

**Results:**

PSMA PET/CT consensus statements concerned utility, best timing for performing, criteria for evaluation of response, patients who could benefit, and handling of radiolabeled PSMA PET tracers. Consensus was reached on all statements. PSMA PET/CT can be used before and after any local and systemic treatment in patients with metastatic disease to evaluate response to treatment. Ideally, PSMA PET/CT imaging criteria should categorize patients as responders, patients with stable disease, partial response, and complete response, or as non-responders. Specific clinical scenarios such as oligometastatic or polymetastatic disease deserve special consideration.

**Conclusions:**

Adoption of PSMA PET/CT should be supported by indication for appropriate use and precise criteria for interpretation. PSMA PET/CT criteria should categorize patients as responders or non-responders. Specific clinical scenarios deserve special consideration.

## Introduction

Prostate-specific membrane antigen (PSMA) positron emission tomography (PET)/computed tomography (CT) is being used more frequently for prostate cancer (PCa) staging and for localization of recurrent disease [1].

International guidelines, including the guideline of the European Association for Urology (EAU) [2], have incorporated PSMA PET/CT and provide recommendation on its appropriate use. It is the consensus imaging modality in patients with rising PSA after radical treatment to confirm a diagnosis of oligorecurrent (metachronous) oligometastatic PCa [3]. In other clinical situations, such as PSA persistence after radical treatment, PSMA PET/CT is also recommended [2], while for initial staging of high-risk patients, the ultimate role of PSMA PET/CT is still debated. The most recent results of prospective trials are very promising [4, 5].

There are procedure guidelines for performing PSMA PET/CT endorsed by European Association of Nuclear Medicine (EANM) [1]. PSMA PET/CT is considered a potentially useful tool for evaluating responses to therapy, but there is lack of data regarding criteria to be used for evaluating PSMA PET/CT findings in relation to therapy response assessment [2, 4]. In recognition of the growing importance of PSMA radiopharmaceuticals in the diagnosis and treatment of patients with PCa, the EAU in collaboration with EANM recruited a panel of international experts in PCa within the fields of nuclear medicine, radiology, and urology, who met on February 21, 2020, in Amsterdam, The Netherlands, for an European consensus panel to formulate criteria for PSMA PET/CT-based response and progression in patients treated for metastatic PCa and when to use it. The main output of this meeting was the development of sets of consensus statements concerning PSMA PET/CT criteria for response assessment in various clinical scenarios for patients with metastatic PCa, and these are presented here.

## Materials and methods

A modified nominal group technique was used to reach consensus statements on PSMA PET/CT response assessment criteria [6, 7]. Ten weeks prior to meeting, each panelist received thematic topics concerning PSMA PET/CT response assessment along with relevant literature suggested by the panelists. The panelists (Stefano Fanti, Karolien Goffin, Boris Hadaschik, Ken Herrmann, Tobias Maurer, Daniela Oprea-Lager, Wim JG Oyen, Olivier Rouvière, and Anders Bjartell), all authors of this paper, are PCa experts in nuclear medicine (five panelists), radiology (one panelist), or urology (three panelists). A one-day meeting was held, where the thematic topics were again presented. The panelists were subsequently divided into two groups, each containing a balance of experts in the aforementioned specialties, to separately discuss these thematic topics in greater detail and develop statements related to the use of PSMA PET/CT. The breakout groups then reconvened, discussed all statements written by each subgroup, and created a combined list of statements organized under thematic topics.

For the scope of the present project, PSMA PET/CT refers to PET/CT imaging performed with PSMA-targeting radioligands (e.g., [^68^Ga]Ga-PSMA-11, [^18^F]F-PSMA-1007, [^18^F]DCFPyL).

Each statement was singular and phrased so that panel members could agree or disagree with it. The resulting statements were uploaded to an online voting tool, and the panelists voted anonymously online [8]. Panelists voted on a nine-point scale, ranging from strongly disagree (1) to strongly agree (9) (i.e., 1–3 disagree, 4–6 uncertain, 7–9 agree). The results of the vote on each statement were conveyed to participants immediately after voting.

The statistical analysis of the voting focused on the level of agreement (median score) with each statement and whether there was consensus (dispersion of scores around the median). This followed the methods proposed by the research and development project (RAND)/University of California, Los Angeles (UCLA) Appropriateness Method which has been shown to be robust for smaller panels and can be used in panels of any size [9]. For each statement, first the median score was calculated, then the 30th and 70th percentiles were calculated, which constitute the interpercentile range (IPR). The IPR was used to calculate the interpercentile range adjusted for symmetry (IPRAS), which is calculated using the formula: IPRAS = 2.35 + (asymmetry index [AI] * 1.5), where the AI is defined as the absolute difference between the central point of the IPR and 5 (i.e., the central point on the 1–9 scale). If the IPR < IPRAS, then this indicates there is no extreme dispersion of scores (i.e., there is “consensus”).

The median agreement score (MAS) was used to determine the level of agreement: a MAS in the 1–3 range indicated the panel disagreed with the statement, a MAS in the 4–6 range indicated the panel were uncertain, and a MAS in the 7–9 range indicated the panel agreed with the statement. Then, the IPRAS was used to assess consensus. For a statement to be considered to have agreement and consensus, two conditions had to be met: a median score in the 7–9 category and an IPRAS < IPR.

## Results

Within the scope of each thematic topic, statements were developed by the panelists during the one-day meeting concerning how to interpret PSMA PET/CT to define response and progression in patients treated for metastatic PCa and when to use it. These thematic topics and statements along with the corresponding MAS, IPR, and IPRAS from the voting are reported in Table 1. Briefly, the statements covered the best timing for performing PSMA PET/CT, the criteria that should be used to evaluate the PSMA PET/CT response, the patients who could benefit from such evaluation from a clinical perspective, and how the various PSMA-targeting PET tracers should be handled.

**Table 1:** Consensus statements on PSMA PET/CT response assessment criteria along with their respective median agreement scores (MAS), interpercentile ranges (IPR), and interpercentile ranges adjusted for symmetry (IPRAS)

Thematic topic and corresponding statement				
Statement		MAS	IPR	IPRAS
1. Can PSMA PET/CT be used before and after any treatment?				
1.1	In principal, PSMA PET/CT can be used before and after any local and systemic treatment in patients at risk of having metastatic disease (including N1 disease).	8	1	7.6
1.2	PSMA PET/CT should not be used if no change of clinical management is expected.	9	0	8.35
1.3	PSMA PET/CT should not be used if local disease within the prostate is expected.	8	1	7.6
1.4	After salvage treatment of oligometastatic PCa with curative intent, if PSA indicates complete response, PSMA PET/CT is to be avoided as an assessment tool.	9	0.6	7.9
1.5	Routine use of PSMA PET/CT should not be used in patients with hormone sensitive PCa and PSA response under systemic therapy.	8	0.6	7.3
1.6	Routine use of PSMA PET/CT should not be used in patients with late-stage disease where no further therapies can be initiated.	7	0	5.35
2. What is the best timing for performing PSMA PET/CT?				
2.1	In cases where the primary PCa is negative on PSMA PET/CT, further staging with PSMA PET/CT is generally not adequate, and other imaging should be performed.	9	0	8.35
2.2	If used for response assessment, then a PSMA PET/CT should be performed before the start of treatment (i.e., baseline PSMA PET/CT).	9	0	8.35
2.3	PSMA PET/CT should not be performed within three months after initiation of systemic therapy in hormone sensitive PCa.	9	1	7.6
2.4	PSMA PET/CT should not be performed for routine follow-up if management of the patient is unlikely to change.	9	0.6	7.9
3. Which PET criteria should be used to evaluate the PSMA response?				
3.1	For clinicians, PSMA PET/CT should always be accompanied by evaluation of clinical and laboratory data.	9	0	8.35
3.2	For imaging specialists, only treatment response criteria for PSMA PET/CT are needed (not taking other parameters into account (e.g. PSA, etc.).	9	1	7.6
3.3	Ideally, PSMA PET/CT criteria should categorize patients as responders or non-responders.	9	0	8.35
3.4	Categories of responders should include patients with stable disease and partial and complete response on PSMA PET/CT imaging; non-responders should include patients with progressive disease on PSMA PET/CT.	8	1.2	6.85
3.5	In early recurrent PCa, appearance of any new lesion with high suspicion should be regarded as progressive disease.	8	1	7.6
3.6	In polymetastatic PCa, increase of uptake or tumor volume > 30% is defined as progressive disease.	8	1	7.6
3.7	In polymetastatic PCa, appearance of two or more new lesions should not be regarded as progressive disease, if total tumor volume or uptake does not increase > 30%.	9	1	7.6
3.8	PCWG3-criteria for evaluation of bone scintigraphy should not necessarily be used for PSMA PET/CT evaluation.	9	0.6	7.9
3.9	Complete response: complete disappearance of any lesion with tracer uptake; partial response: reduction of uptake and tumor volume by > 30%; SD: change of uptake and tumor volume ± ≤ 30% and no new lesions; progressive disease: appearance of two or more new lesions and/or increase of uptake or tumor PET volume > 30%.	9	0.6	7.3
4. Which patients could benefit from PSMA response assessment from a clinical perspective (other than those for whom a treatment change will have a significant impact on outcome)?				
4.1	PSMA PET/CT response assessment should be evaluated in the context of clinical trials.	9	0	8.35
4.2	In clinical practice, PSMA PET/CT response assessment may be performed in patients with inconsistent laboratory findings and/or clinical course of the disease if change of management is considered.	9	0	8.35
5. How should the various PET tracers be handled?				
5.1	Different ^68^Ga and ^18^F radiolabeled PSMA tracers (e.g., [^68^Ga]Ga-PSMA-11, [^18^F]PSMA-1007, and [^18^F]DCFPyL) show similar performance even if there is lack of comparative data.	8	1	7.6
5.2	For response assessment the same PSMA PET tracers should be used.	9	0	8.35
5.3	Quality assurance is mandatory either for radiotracer production and image acquisition and should include EARL-harmonized protocols (scanner, reconstruction algorithms) regarding dosage, time of acquisition and quantification should be done using validated software.	9	0	8.35
6. Other relevant statements				
6.1	Available real-world data and/or trial data should be analyzed for response assessment with PSMA PET/CT before designing future prospective trials.	9	0	8.35
6.2	Criteria for treatment assessment should be evaluated in test-retest trials to assess normal variability in the measurement.	9	1	7.6

*EARL*, European Association of Nuclear Medicine Research Ltd.; *IPR*, interpercentile range; *IPRAS*, interpercentile range adjusted for symmetry; *MAS*, median agreement score; *PCa*, prostate cancer

Other relevant thematic topics were also addressed with statements: Available real-world data and/or trial data should be analyzed for response assessment with PSMA PET/CT before designing future prospective trials; Criteria for treatment assessment should be evaluated in test-retest trials to assess normal variability in the measurement. In every instance, the MAS fell in the 7–9 category and IPR as lower than IPRAS, so there was “agreement” and “consensus.”

As an antecedent to the discussion of the thematic topics, the panelists considered the utility and impact of PSMA PET/CT in PCa and developed four statements and reached consensus on all four.

• PSMA PET/CT is currently the most sensitive imaging staging technique for depicting metastases at any stage of the disease. MAS = 8 (IPR = 1; IPRAS = 7.6).

• Use of PSMA PET/CT will cause stage migration, which is a problem for clinical decision-making. MAS = 8 (IPR = 0; IPRAS = 6.85).

• PSMA PET/CT should be used as imaging tool at biochemical recurrence. MAS = 9 (IPR = 0; IPRAS = 8.35).

• PSMA PET/CT should not be used in low-risk disease. MAS = 9 (IPR = 0; IPRAS = 8.35).

## Discussion

All consensus statements were developed following the European Society for Radiotherapy and Oncology (ESRO) and European Organisation for Research and Treatment of Cancer (EORTC) consensus recommendations for classification of oligometastatic disease [10].

### General statements

Regarding the use of PSMA PET/CT for assessing response to therapy, it was confirmed that such an approach should be used only if a change of clinical management is expected from the results (statements 1.2 and 2.4) as already emphasized in EAU Guidelines [2]. Thus, in case of good PSA response after salvage treatment, there is no indication for performing PSMA PET/CT (statement 1.4); also, in case of late-stage disease where no further therapies can be initiated or hormone sensitive PCa with favorable PSA response under systemic therapy, there is no role for PSMA PET/CT (statements 1.5 and 1.6).

Asking whether PSMA PET/CT can be used before, after, and during treatment is an important question, because there are, for example, concerns regarding how to interpret uptake of tracer especially in hormone-naive patients starting androgen deprivation therapy (ADT) [11]. Consensus was reached on the statement that in principle, PSMA PET/CT can be used before and after any local and systemic treatment in patients with metastatic disease (including N1 disease). PSMA PET/CT is actually the most sensitive technique to detect metastases, and thus, it is likely that the prognosis of M0 and M+ patients based on PSMA PET/CT will be changed, as compared to the prognosis of M0 and M+ patients based on standard work up (CT and bone scan). Nonetheless, in the absence of clinical trials assessing the prognosis of patients with metastases detected only by PSMA, it remains unclear whether PSMA PET/CT should be widely used in high-risk patients.

The panel is well aware that the proposed criteria are based on practical experience and authors’ opinions; indeed, there is very little data to ultimately support some of the statements that are made. Nonetheless, it is clear that some baseline criteria are proposed to be used for future studies and applied for clinical scenarios that are already happening. With expected publication of more data from prospective studies, it will hopefully be possible to refine the statements proposed here, possibly with future further consensus events to be held for review of datasets with outcome measures. Once the criteria have been evaluated in clinical trials, they could surely be improved.

### Timing for PSMA PET/CT

With respect to the best timing for performing PSMA PET/CT, some evident prerequisites were confirmed, such as that in case of primary PCa negative on PSMA PET/CT, further imaging with PSMA PET/CT is generally not adequate (statement 2.1), and if used for response assessment, baseline PSMA PET/CT should be performed before the start of treatment (statement 2.2).

The panelists agreed that potential flare observed in a PSMA scan following initiation of ADT is an early event, and misinterpretation can be avoided by performing the scan not earlier than 3 months after the start of ADT (see also statement 2.3). Data concerning the flare phenomenon observed with ADT and second-generation anti-androgen therapy are limited and not unequivocal [12–15]. The correlation between androgen signaling and PSMA expression seems to be quite complex and requires more study before practice-defining conclusions can be drawn. Nonetheless, the panelists, based on their practical experience, suggested 3 months as the minimal timing for performing PSMA PET/CT after initiation of hormonal intervention. A prospective study has shown that androgen deprivation therapy could impact the timing for PSMA PET/CT [16]. The study included 15 men, eight patients with metastatic hormone sensitive PCa treated with luteinizing hormone-releasing hormone and bicalutamide and seven patients with castration resistant PCa treated with enzalutamide or abiraterone, and showed that at 9 days post treatment, [^68^Ga]Ga-PSMA-11 PET/CT SUVmax decreased in the hormone-sensitive patients (median 30% (interquartile range 5–61) but increased in the castration-resistant PCa (median 45% (interquartile range 12.7–66)).

### Criteria for interpretation of PSMA PET/CT

Criteria to be used to evaluate PSMA response were thoroughly discussed and addressed. Indeed, molecular response criteria have been proposed as better than morphological criteria in patients with metastatic castration resistant PCa [17], but the precise criteria to be used have not been identified. There are no clear definitions for oligometastatic and polymetastatic disease, so, in the context of this report, progression was defined in order to start creating guidance on how to use PSMA PET/CT. In general, there is confidence in drawing conclusions in cases with complete response as well as for detecting clear progression of disease and scoring of new lesions [18–20], but uncertainty remains for everything in between.

The panelists agreed that PSMA PET/CT criteria should categorize patients as responders or non-responders (statement 3.3) for both, clinical use and trial incorporation. Given the nature of PCa, it was agreed that categories of responders should include patients with stable disease, partial response, and complete response on PSMA PET/CT imaging; non-responders should include patients with progressive disease on PSMA PET/CT (statement 3.3). In this light, the consensus statements were written with progression defined as a 30% increase of tumor burden, and this is in line with other studies and modified PET response criteria in solid tumors (PERCIST) [21–23]. In general, the panel agreed on a definition of PSMA response as Complete in case of disappearance of any lesion with tracer uptake; Partial as reduction of uptake and tumor PET volume by > 30%; Stable as change of uptake and tumor PET volume ± ≤ 30% without evidence of new lesions; Progression as appearance of > 2 new lesions or increase of uptake or tumor PET volume >= 30% (statement 3.9).

While these criteria are ready to be used in most situations, and should be evaluated in future studies, some specific clinical scenarios deserve special consideration. The definition of progression indicated by the panel is largely in line with the proposal of PSMA PET progression [18], largely based on the appearance of ≥ 2 new lesions, which is adequate in many cases. Nonetheless, the panelists agreed that in early recurrent PCa, appearance of any new lesion with high suspicion should be regarded as progressive disease (statement 3.5). The rationale was to maximize the sensitivity of the approach in such settings, where additional local therapies may still be proposed. Conversely, in polymetastatic PCa, increase of uptake or tumor PET volume > 30% was regarded as progression; appearance of two or more new lesions should not be necessarily regarded as progressive disease, if total tumor PET volume or uptake does not increase > 30% (statements 3.6 and 3.7). For the purpose of this project, the definition of a new lesion is based on the appearance of a new focal area of PSMA uptake at PET, with or without CT change. Disease progression should not be based solely on PET/CT. The rationale here was to avoid misinterpretation of progressive disease in case of an overall good response of known lesions to systemic therapy with only appearance of a few minor lesions. Indeed, it would be a mistake to switch an active treatment too early in polymetastatic situations.

Semi-quantitative evaluation of radiolabeled PSMA PET/CT scans could be very useful when assessing response to treatment. Standardized uptake value (SUV) is the most commonly used semi-quantitative parameter, providing a measure of radiotracer uptake. The main recommendation for using SUV parameters, (SUVmax, SUVmean, or SUVpeak) is to optimize reproducibility by limiting all variation factors (quality of injection, uptake time, type of scanner, reconstruction algorithm, and others), namely to rigorously harmonize procedures and parameters in all scans. Volumetric PET measurements (so called tumor burden values) based on available software packages can partly overcome such problems [24, 25], since they account for less variations across patients and are better reproducible. However, these parameters are still not widely used in clinical practice because they are technically demanding. Tools for quantitative estimation of the total tumor burden are under development, and these could reduce current problems with interobserver variability and low reproducibility with manual interpretation of images.

The use of uptake thresholds based on PERCIST could not be supported by the literature, because these have only been validated for FDG PET. Nonetheless, the panelists agreed that a threshold should be arbitrarily chosen as baseline for future studies; furthermore, it should be at least wider than repeatability coefficients (published only for the PSMA-targeted agent DCFPyL) and could be modified in the future to hopefully arrive at the appropriate cut-offs for tumor response [25].

### Need for standard interpretation of scans

Quality standards for interpretation of PSMA PET/CT scans are needed. Visual, qualitative assessment of the attenuated and non-attenuated PET images is the basis of any PET study interpretation. The major concern regarding this assessment is the significant inter- and intra-observer variability of PET image interpretation. Therefore, quality standards for interpretation of PSMA PET/CT scans are needed. Yet, the report from the nuclear medicine physician needs to adhere to quality standards, as recently proposed in the E-PSMA: the EANM standardized reporting guidelines for PSMA PET/CT. The quality of the procedure itself is standardized using existing procedures guidelines to obtain high-quality imaging [1], and the same applies to quantification software.

Different PSMA tracers, including [^68^Ga]Ga-PSMA-11 and [^18^F]F-labeled PSMA-1007 and [^18^F]DCFPyL tracers, are supposed to perform similarly, even if there is lack of comparative data [26]. For response assessment, it is evident that the same PSMA PET tracers should be used for baseline and subsequent imaging. Quality assurance is mandatory for both radiotracer production and image acquisition.

### Limit of the methodology

There was consensus for every statement, and this is undoubtedly related to the fact that the panel mostly created the statements on the meeting day and discussed them in depth before voting. In-group bias is therefore a potential limitation, but the panel were sampled from the two most directly relevant professional societies (EANM and EAU) and these individuals represent expertise on this topic. Furthermore, voting was anonymous, so had any participant disagreed with the statements, there was a channel to express disagreement.

The panelists refrained from evaluating response to some particular therapies due to lack of data. As previously mentioned, the proposed criteria were mainly based on practical experience. Lutetium PSMA, due to its theranostic nature, is the only treatment where PSMA PET/CT has been used more frequently to evaluate response to therapy [27, 28]. For this indication, PSMA PET/CT is mandatory for establishing the suitability of treatment, and prognostic information can be derived from baseline PSMA PET/CT [29]. It is also possible, that PSMA PET/CT could be used to describe duration of response and decide the frequency of treatment.

### Perspective

Available real-world data and/or trial data should be analyzed for response assessment with PSMA PET/CT before designing future prospective trials. Furthermore, criteria for treatment assessment should be evaluated in test-retest trials to assess normal variability in the measurement.

## Conclusions

PSMA PET/CT is currently the most rapidly growing imaging technique in PCa, but its adoption should be adequately supported by indication for appropriate use and precise criteria for interpretation. PSMA PET/CT holds great potential in several clinical situations. Use of PSMA PET/CT should not be considered, if no change of clinical management is expected.

For evaluation of response to treatment, PSMA PET/CT can be used before and after any local and systemic treatment in patients with metastatic disease. However, it should not be performed within 3 months after initiation of systemic therapy in hormone sensitive PCa.

Ideally, PSMA PET/CT criteria should categorize patients as responders or non-responders: categories of responders include patients with stable disease, partial response, and complete response on PSMA PET/CT imaging. Specific clinical scenarios such as oligometastatic or polymetastatic disease deserve special consideration. The use of PSMA PET/CT response assessment should be implemented and evaluated in the context of clinical trials.

EAU and EANM endorse and promote high-quality standards in performing and reporting PSMA PET/CT scans.
